# Efficacy of Upadacitinib in a Middle‐Aged Man With Netherton Syndrome Refractory to Dupilumab: A Case Report

**DOI:** 10.1111/1346-8138.70214

**Published:** 2026-03-07

**Authors:** Bo‐Jie Yu, Pei‐Ling Li, Bing‐Sian Lin, Shou‐En Wu, Chih‐Tsung Hung

**Affiliations:** ^1^ Department of Dermatology Tri‐Service General Hospital, National Defense Medical University Taipei Taiwan; ^2^ Department of Medical Research Tri‐Service General Hospital, Graduate Institute of Medical Sciences, National Defense Medical University Taipei Taiwan

Dear Editor,

1

Netherton syndrome (NS) is a rare, autosomal recessive disease caused by mutations in *SPINK5*. Typical presentations include congenital skin and hair abnormalities and atopic manifestations [1]. We present a patient with NS responsive to upadacitinib 30 mg with sustained efficacy.

A 51‐year‐old Han Chinese man with a lifelong history of atopic dermatitis (AD) had failed multiple treatments, including topical corticosteroids, oral cyclosporine, methotrexate, and phototherapy (Figure 1a). Given limited efficacy and high serum immunoglobulin‐E (IgE) (1759 IU/mL), he began dupilumab (600 mg loading dose, then 300 mg every 2 weeks). At baseline, body surface area (BSA) involvement was 40%, Eczema Area and Severity Index (EASI) score was 20.7, and Investigator's Global Assessment (IGA) score was 3. However, his condition worsened, and we prescribed systemic corticosteroids, antihistamines, and empirical antibiotics. Two weeks after the fifth dose, he developed generalized, painful erythroderma with double‐edged scales and erosions, involving 83% of BSA despite decreased IgE (399 IU/mL) (Figure 1b).

**FIGURE 1:**
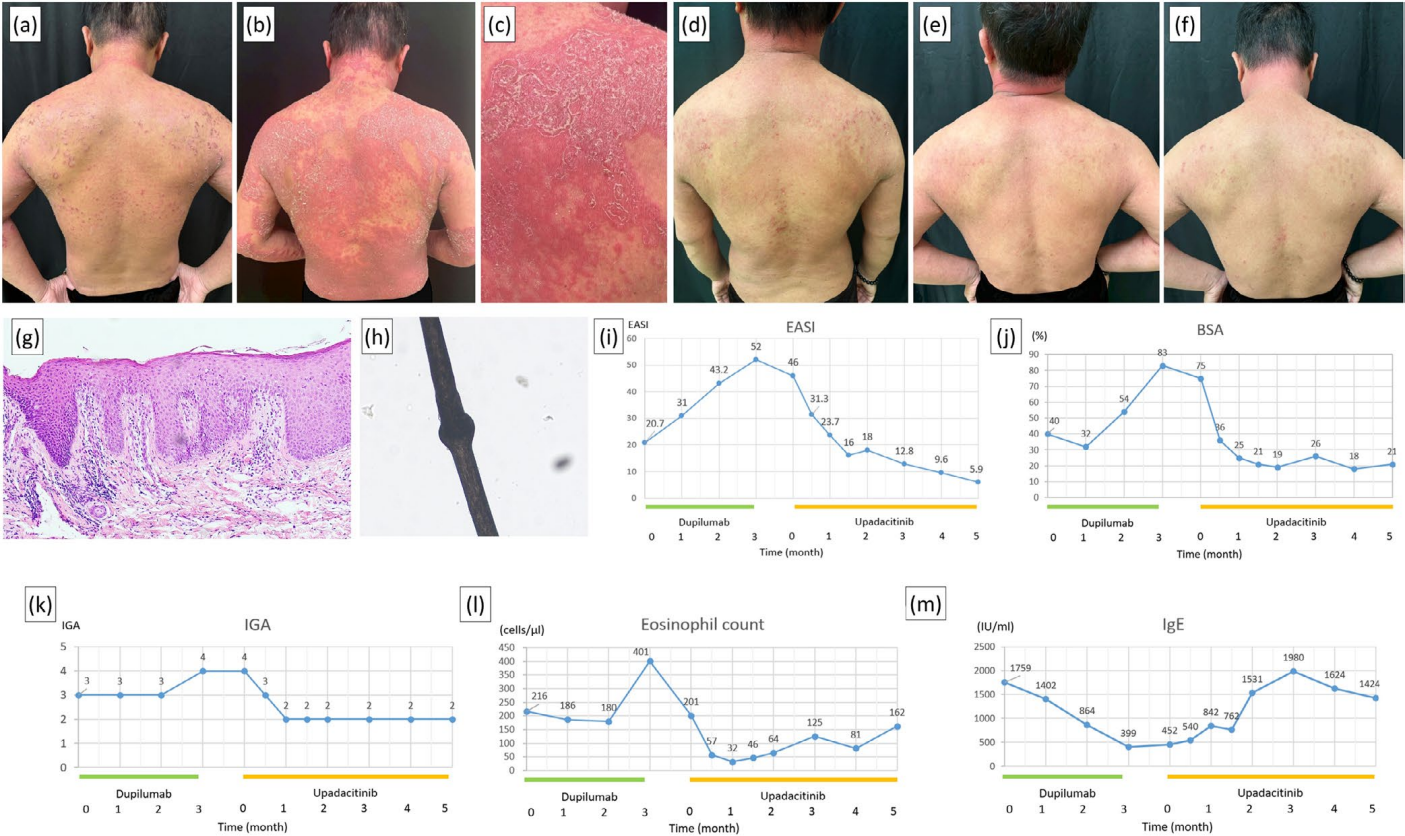
The clinical features and measurements during dupilumab therapy and upadacitinib therapy of the patient. (a) The clinical features of the patient before dupilumab treatment. (b) After 12 weeks of dupilumab treatment, the patient exhibited generalized, erythematous to dark‐red patches and plaques with double‐edged scales along with erosions, and (c) the close‐up view of right upper back revealing ichthyosis linearis circumflexa. (d) After 2 weeks of upadacitinib treatment, a significant decrease in erythema and scaling is observed. (e) After 6 weeks of upadacitinib treatment. (f) After 5 months of upadacitinib treatment, the patient achieved sustained remission with residual post‐inflammatory hyperpigmentation. Improved hair texture and visible regrowth were observed compared to baseline. (g) Skin biopsy revealed non‐specific parakeratosis, psoriasiform epidermal hyperplasia and elongated rete ridges (hematoxylin and eosin (H&E), magnification × 100). (h) Trichorrhexis invaginata under light microscopy (magnification × 100). (i–k) Eczema Area and Severity Index (EASI), Body surface area (BSA) involvement, and Investigator's Global Assessment (IGA) scores increased during dupilumab therapy, and then decreased over 5 months of upadacitinib treatment. (l) Eosinophil counts elevated during the period of erythroderma (3 months after dupilumab treatment) before returning to baseline. (m) Serum IgE levels decreased during dupilumab treatment and returned to baseline levels following upadacitinib therapy.

Considering exacerbated erythroderma, dupilumab was discontinued. Skin biopsy showed nonspecific parakeratosis and psoriasiform epidermal hyperplasia (Figure 1g), and differential diagnosis included psoriasis and psoriasiform dermatitis. However, the presence of double‐edged scales raised suspicion of ichthyosis linearis circumflexa (ILC) (Figure 1c). Trichoscopy revealed trichorrhexis invaginata (Figure 1h), and whole‐exome sequencing detected compound heterozygous variants in *SPINK5* (c.80A>G and c.2423C>T), confirming NS.

Four weeks after discontinuing dupilumab with presence of erythroderma, the patient started oral upadacitinib (30 mg daily) alongside topical diflucortolone. Baseline infection screening was negative for tuberculosis and hepatitis. After 2 weeks, erythema remarkably decreased (Figure 1d). After 6 weeks, BSA improved to 21% and IGA improved to 2 (Figure 1e). The EASI score declined, but serum eosinophil count and IgE returned to baseline (Figure 1i–m). This efficacy has been sustained for 5 months without significant side effects (Figure 1f), maintaining normal liver function and lipid profiles.

In NS, dysregulated kallikrein‐related peptidase 5 drives Th2 differentiation, yet dupilumab shows variable efficacy [1]. This may stem from complex Th17 and interferon pathway involvement [2]. While previous literature associated ILC and scaly erythroderma with different immune profiles [1], our patient's erythroderma with features of ILC might reflect possibilities of overlapping NS‐spectrum phenotype or shifting inflammatory pattern during treatment. Alternatively, natural disease progression or infection from barrier dysfunction may contribute to exacerbation.

Upadacitinib, a selective Janus kinase 1 (JAK1) inhibitor, blocks the JAK–STAT and interferon pathways. Although previous reports showed promising results in the first month, some cases were discontinued within 4 months due to waning efficacy or adverse effects [1, 3, 4]. Previous studies disclosed 30 mg upadacitinib for moderate‐to‐severe AD might achieve optimal disease control [5]. Considering disease severity and national health insurance reimbursement criteria, we initiated treatment at 30 mg daily, resulting in sustained clinical improvement for 5 months. Although optimal dosing strategies need to be established, increased infection risks remain a concern.

In conclusion, we present severe NS‐spectrum dermatitis refractory to dupilumab improved with JAK1 inhibition, providing a perspective for treating refractory NS. Further research with longer follow‐up is essential to establish long‐term safety and effectiveness.

## Ethics Statement

The patients in this manuscript have given written informed consent to the publication of their case details. Reviewed and approved by Institutional Review Board of Tri‐Service General Hospital (TSGHIRB No.: B202515176, approval date: Oct 31, 2025).

## Conflicts of Interest

The authors declare no conflicts of interest.

## Data Availability

The data that support the findings of this study are available from the corresponding author upon reasonable request.
